# Supplementary material for: The benefits of action to reduce household air pollution (BAR-HAP) model: A new decision support tool
Source: PLoS One. 2021 Jan 22;16(1):e0245729. doi: 10.1371/journal.pone.0245729 (PMC7822293; doi:10.1371/journal.pone.0245729)
Supplement: Attachment — Submitted filename: PLOS One_BAR-HAP Tool_Response to Reviewers_final.docx [file pone.0245729.s001.docx]

**Manuscript #:** PONE-D-20-17182

**Manuscript Title:** The benefits of action to reduce household air pollution (BAR-HAP) model: A new decision support tool for energy and health sector decision-makers

**Response to Reviewers**

We would like to thank the editor and reviewers for their reading and positive comments on our manuscript. We have responded to the helpful suggestions for improvement, and believe that the manuscript is consequently considerably improved. Our point-by-point responses are in blue text below.

Editor’s additional requirements

1. Please ensure that your manuscript meets PLOS ONE's style requirements, including those for file naming. The PLOS ONE style templates can be found at

[https://journals.plos.org/plosone/s/file?id=wjVg/PLOSOne_formatting_sample_main_body.pdf](https://urldefense.com/v3/__https:/journals.plos.org/plosone/s/file?id=wjVg*PLOSOne_formatting_sample_main_body.pdf__;Lw!!OToaGQ!8aOvEVVJMKJzluBNGgy3r4v3SLHzj8-rPO3IearoZK7sqqwo5X0R6uEZnG6Hly7IPLA$) and <https://journals.plos.org/plosone/s/file?id=ba62/PLOSOne_formatting_sample_title_authors_affiliations.pdf>

Response: Thank you for pointing us to the PLOS ONE style templates. We have revised our manuscript to meet PLOS ONE’s requirements, but have numbered each section and sub-section to help the reviewers.

2.We note that you have stated that you will provide repository information for your data at acceptance. Should your manuscript be accepted for publication, we will hold it until you provide the relevant accession numbers or DOIs necessary to access your data. If you wish to make changes to your Data Availability statement, please describe these changes in your cover letter and we will update your Data Availability statement to reflect the information you provide.

Response: In the revised manuscript, we have provided the [web link](https://www.who.int/airpollution/household/interventions/chest-module3-BAR-HAP-tool/en/#:~:text=The%20WHO%20Benefits%20of%20Action,cooking%2Drelated%20household%20air%20pollution) to the published BAR-HAP Excel tool and the corresponding user’s manual that includes all model equations. Readers can now access the data sources cited in the manual or access those used in the model itself.

3. Please include captions for your Supporting Information files at the end of your manuscript, and update any in-text citations to match accordingly. Please see our Supporting Information guidelines for more information: [http://journals.plos.org/plosone/s/supporting-information](about:blank).

Response: Now that we have provided a stable web link for the supporting materials (manual and tool itself), we have removed the supporting information that was duplicative (and this will also allow readers to access the most up to date guidance on the model online).

Reviewer #1

This is a paper that presents a new decision-support tool for assessing the impacts of cleaner cooking fuel on multiple sustainability-relevant goals, including household air pollution. It builds on a prior cost-benefit model published in 2018, which was expanded in this work. The tool was piloted in a case study in Nepal.

While the tool seems appropriate for the problem and builds strongly on other peer-reviewed work, several revisions could make this paper clearer and more well-supported.

1) It is unclear which household air pollutants are accounted for as influencing health, and which as influencing climate. For example, on page 1, black carbon is noted only as a climate forcing agent — is its influence on health also calculated? What about other household air pollutants?

Response: In our calculations we account for (a) PM_2.5_ as influencing the five household air pollution-related health outcomes under consideration (COPD, ALRI, IHD, lung cancer and stroke), and (b) carbon monoxide, carbon dioxide, methane, black carbon, organic carbon and nitrous oxide as influencing climate. Please note that black carbon is included in PM_2.5_, due to its very small size, but, consistent with the environmental health literature, we do not differentiate the health impacts of black carbon versus the other constituents of PM_2.5_. We have clarified these aspects in our revision, on p.14 and in footnotes 8 and 9: “In our calculations we account for PM_2.5_ as influencing the five household air pollution-related health outcomes under consideration (COPD, ALRI, IHD, lung cancer and stroke)” and “parameters related to the global warming potential of relevant pollutants (which include CO2, CO, N2O, CH4, BC and OC, where the latter is a net cooling agent)” and “We account for CO_2_, CO, N_2_O, CH_4_, BC and OC as influencing climate. Please note that black carbon is included in PM_2.5_, due to its very small size, but, consistent with the environmental health literature, we do not differentiate the health impacts of black carbon versus the other constituents of PM_2.5_.”

2) This raises a broader question of the detailed equations, which are missing from the paper. It is stated they will be made available, but it is nearly impossible to do a thorough technical review of this manuscript without this detailed information to benchmark the realism of the simulations.

Response: As noted on p. 8 (footnote 4 and references), the published user’s manual is now available online. This was previously provided as supplementary information to the manuscript, but we apologize if this was not clear. This manual includes the detailed equations for all of the model calculations, including those that correspond to various policy interventions. These equations are consistent with those available in several pre-cursor publications, but have been further developed to incorporate the effectiveness of specific policy interventions, and to allow calculation of health metrics such as disability adjusted life years (DALYs) averted and cost effectiveness metrics related to DALYs avoided.

3) While the tool’s specific domain is Nepal, it is noted that it could be applied elsewhere, and that data will need to be updated. Some more detailed discussion about model limitations (other than data) in applicability to other regions would be useful. To what extent do the fundamental assumptions about processes simulated hold across the world?

Response: The model’s underlying assumptions are largely transferrable across regions, once context-specific data are incorporated into the tool. The only exception to this is in the potential inclusion of additional technology or fuel transitions. A longer-term goal of the collaboration that produced this manuscript is to create the global database that would facilitate calculations across countries, but we felt it important to ensure that the basic structure and aims of the model were peer-reviewed prior to this full expansion, in the event that structural changes were deemed important. The expanded version of the BAR-HAP tool will take into account the need for additional transitions, and allow calculations by geographic region, and comparison across countries and regions, for use by the global health and energy communities.

A discussion of limitations now appears on p.18:

1. “In its current form, the model is static. It thus does not account for dynamics that increase or decrease population, rates of technology adoption and changes in affordability, or the prevalence or incidence rates of diseases over time.
2. For the most part, the tool does not factor in changes in health sector implementation costs as the scale of intervention provision (either economies or diseconomies of scale) increases. Only the stove and fuel costs, and promotion program costs scale based on the number of users targeted.
3. While we include an exposure adjustment factor, we do not incorporate structural characteristics of the household (for which data may not exist at the country level), thereby not considering cross-household heterogeneity.
4. The contribution of ambient air pollution, which could nullify the effects of HAP reductions from clean cooking, is not accounted for.
5. Consumers’ preferences for improved and clean cooking scenarios and related policy interventions are not incorporated. A welfare-theoretic perspective on private benefits, for example, would equate these to the area under the demand curve, but BAR-HAP calculations of these private benefits are rather based on valuation equations that pertain to the specific benefits presented in Table 1. These may diverge from individuals’ willingness to pay for those improvements for a range of reasons.
6. Though their addition would not be a simple task manageable by the majority of users, additional transitions between cooking fuels and technologies could be incorporated into the tool, which is currently limited to the sixteen described above, and as the piloting in Nepal revealed, users in specific contexts are likely to have particular interest in certain types of transitions that may not be included in the current version.”

We have added language concerning the future expansion plans for the tool to the closing portion of the manuscript, see p. 27:

“WHO is now working with researchers to expand the database underlying the BAR-HAP Tool to facilitate national and global analyses of policy interventions to spur clean cooking transitions. As part of this expansion, additional training workshops will be organized to catalyze informed decision-making processes, and to support the formulation of new policies appropriate for the countries most affected by the negative health impacts of HAP. In this respect, the calculation of context-specific costs and benefits at household and system levels using national and local data is a key strength that will facilitate tailoring of solutions according to the needs and priorities of particular countries.”

4) In the case presented of Nepal, it is unclear whether this is designed as an experimental setting or not, and whether the paper intends to present results from this experiment. If this is the case, more formality in data collection and what questions were tested in this experiment would be useful.

Response: The analysis is not from an experimental setting and our work did not involve primary data collection efforts, so we do not include a formal description of the data collection strategy. As noted in the user manual and revised manuscript, we relied on secondary data to parameterize the model for this setting. Please refer to “Section 3.8 Data Sources” of the manuscript.

5) Relatedly, the paper does not seem to ask and answer a relevant scientific question related to the tool, so it’s unclear what exactly the scientific contribution of this work is, and to which field. Is this an advance on the decision-support tool itself, or the methodology? This could be made much clearer in a revision.

Response: The article is meant to present the decision-support tool and methodology as the scientific contribution. The Nepal application is intended to provide an illustration of the tool, rather than a full policy analysis for that setting. We have clarified these aspects in our revision, in both the introduction and discussion sections. We have also added language to make clearer why we think this tool is needed and useful for decision-makers. The key excerpts are provided below:

p.4: “This paper’s scientific contribution is first, to describe the underlying structure and methods incorporated into the BAR-HAP Tool. Second, it presents an illustrative application of the tool that demonstrates the model’s potential usefulness, for practitioners, in providing a holistic appreciation of the implications of inefficient cooking technology, and for assessment of the pros and cons of different intervention strategies to address this energy poverty problem.”

Pp.25-26: “This paper’s main contribution is in presenting a new decision-support model – the BAR-HAP Tool – that is aimed at helping health and government decision-makers to plan policy interventions to accelerate transitions towards cleaner cooking technologies, and in describing the tool’s methodology. The conceptual model behind the tool is based in a framework of costs and benefits, that accounts for partial adoption and use of technology, and incorporates evidence from recent related modeling efforts and field studies. It allows analysis of pricing, financing, and behavior-change interventions, based on published evidence on the effectiveness of these policy instruments. Practical aspects of the tool were modified based on feedback from a diverse stakeholder workshop held with representatives from the national government, local researchers, professionals working in the health system, and development partners working on health and energy issues in Nepal. We also demonstrated here how the tool can be used to consider several cooking transitions in the context of Nepal, to provide a perspective on the types of results that BAR-HAP can generate.”

Reviewer #2

The paper deals an interesting issue, i.e. cooking with polluting and inefficient fuels and technologies, very interesting especially in the investigated area, Nepal. The authors presented a new model, BAR-HAP, for evaluating and considering the health, environment and development implications in such topic. The approach followed by authors is good, really persuasive, well-reported and presented, following all the research and publication ethics. I suggest to stress or show a comparison with different situations worldwide, to deep the effects on the air quality, to highlight the possibility to export this approach in other situations, to expand the conclusions (they are very poor, 5 lines!).

Response: Thank you for your comments. As mentioned in our response to Reviewer#1, we plan to expand the BAR-HAP tool to allow the type of analysis that the reviewer is requesting, but felt it was important to ensure that the basic structure and aims of the model were peer-reviewed prior to this full expansion. We will work in the future to incorporate data from 134 low- and middle-income countries (building on that currently included for Nepal) to allow comparative analyses of the type the reviewer has in mind. While the model’s underlying assumptions will be the same, we will include country-specific data wherever available, as noted in our response to R1’s third comment.

We have strengthened the discussion and conclusions sections in response to this comment, highlighting the plans for expansion and, in the methods section (p.18), the limitations of the current model.

Reviewer #3

Comments for Manuscript titled: The benefits of action to reduce household air pollution (BAR-HAP) model: A new decision support tool for energy and health sector decision-makers
The manuscript is an illustrative work to showcase the functional characteristics, usability and limitations of the existing Household Air Pollution (BAR-HAP) Tool developed by WHO. The work can be informative and helpful to someone who wants to explore the model in their respective region of study. The model has been explained elaborately with a pertinent case study of Nepal. However, there are some minor corrections before it can be accepted for publication, mostly the readability of the manuscript. There is also a need to revise the textual flow as the organic linkage of statements to each other is missing at some places. Please go through the following comments and address them carefully.
1. Section 1 Introduction: Please correct typographical errors and add missing information in the manuscript. A few instances listed below-
Page 1 “eliminate the use unprocessed coal…” .
Page 5 Conceptual framework –Please expand and define “DALYs”

Response: Thank you for pointing out these errors. We have made these and other corrections in the revised manuscript, following a careful proofreading.

2. Please provide information of the study area and its important climatic and geographical characteristics

Response: We have provided more context on the Nepal case to help the reader interpret our findings. Please see the added paragraph on p. 16, sub-section ‘3.6 Model piloting and testing’.

3. Information regarding types of biomass fuels used in the study area is missing

Response: The aforementioned section introducing the study area now includes information on types of biomass fuels used in Nepal.

4. Section 3 Methods - is too lengthy and requires sub sections to improve readability. Please add sub headings such as Policy instruments, Model assumptions etc.

Response: Thank you for these suggestions. We have included sub-sections in the ‘Methods’ section of the revised manuscript. We have also streamlined the section as far as possible.

5. A separate section is needed to give brief information about the clean cooking interventions and performance metrics

Response: In the ‘Methods’ section (Section 3), under ‘Conceptual framework’ (Section 3.1), we have provided a short description of the various improved and clean cooking interventions under consideration. We have also briefly described the net benefits, cost effectiveness, and public cost metrics that the tool produces. Please refer to Pp. 8-9 in the revised manuscript.

6. The results section should be clearly demarcated as done for other sections

Response: We have now included a ‘Results’ heading in the revised manuscript.

7. What about model validation? Is there any option for that? If not, it should be clearly mentioned in the paper.

Response: We are unsure how the model would be validated, as it is meant to make future predictions pertaining to interventions that have not previously been implemented. Such validation may become possible in the future, following country action, but for the time being, the model is hypothetical and parameterized for a single country, Nepal.

8. “Despite this, total social net benefits are only slightly reduced relative to the LPG only transition, because the natural draft biomass stove still provides significant fuel and time savings, and climate mitigation, previously shown to be important in Figure 3. Benefits can also be generated at somewhat lower subsidy levels, owing to the lower cost of the natural draft stoves.”
Observation regarding climate mitigation potential of natural draft stoves is not correct. Studies have shown that these stoves BC even higher than traditional cookstoves.

Response: While the reviewer is correct that some biomass ICS do have higher BC emissions than traditional stoves, such stoves on average still produce lower climate-forcing emissions, for several reasons. First, they reduce biomass consumption, such that any unsustainable harvesting of firewood (which is the norm in most low- and middle-income countries, see Bailis et al. 2015) leads to lower net CO_2_ emissions. Second, because fuel consumption decreases, even though the ratio of BC to other PM tends to increase, the overall amount of BC may also decline. Of course, this is not always the case, and there is considerable variation across biomass ICS, but our calculation relies on averages across a number of different models, which on average are helpful in reducing overall BC emissions and climate forcing. If users were interested in particular ICS technologies, the net reductions might not hold, depending on these fuel efficiency and BC emissions characteristics. We have added nuance to explain this in our revision, see p. 24, footnote 12.

9. “Furthermore, users should understand the limitations of the tool, discussed in this article, including the fact that it only covers the contribution of cooking practices to HAP (and does not cover lighting and heating, for example).”
There are a few other limitations that should be admitted in this paper:
i) The exposures vary widely depending on the structural characteristics of household, type of solid biomass fuels used and user behaviour which I am not sure has been incorporated in the model. These variations may induce appreciable changes in the overall exposure of the user to the emissions and eventually the health outcomes. This should be added to limitations.

Response: Thank you for your comments on the limitations of our analysis. While we do not incorporate structural characteristics of the household (the data for which may not exist at a country level), we include an exposure adjustment factor to account for the difference between kitchen concentrations and user exposures from a recent meta-analysis of such data (Pope et al. 2020). This adjustment accounts for aspects such as behavior and housing structure, as well as some stove stacking (i.e. simultaneous use of multiple stoves-traditional and improved). Still, we have noted in our revision that we do not consider cross-household heterogeneity due to factors such as behavior and housing structure.

ii) Indoor air quality concentrations are also impacted by ambient air quality of the immediate locality which can sometimes nullify the reductions brought about by the use of clean cooking in devices. The immediate air pollutant levels high also due to partial uptake of clean cookstoves in a village for example, while most the households are using traditional cookstoves which allows emissions to get released into the outside environment. This also been ignored in the model.

Response: We have noted this limitation in our revision.

10. The modifications done in the model with respect to area of study should be clearly demarcated and explained so that it is clear to the reader how much the results can vary through such modifications.

Response: Please refer to our response to reviewer 1 comment 3. The main limitation with regards to study location pertains to the transitions considered, and the points mentioned above in your comment 9, which are now acknowledged in our revision.

11. Quality of the figures can be improved with better resolution.

Response: Thank you for your feedback. We have revised the figures.

Reviewer #4

The benefits of action to reduce household air pollution (BAR-HAP) model: A new
decision support tool for energy and health sector decision-makers

The authors present a novel decision tool, to help policy makers related to energy, be able to project the effect of different policies on health outcomes along with the cost benefit analysis. There are not many decision tools available in resource limited setting to help decide on the policy selection. Though similar framework had been used to evaluate effects of intervention in Bangladesh. Some suggestions

1. Title could be shortened

Response: Thank you for your suggestion. We now have a shorter title.

2. Introduction could be shortened

Response: We have revised the Introduction section in the revised manuscript, but feel that the material provided there now is largely appropriate and useful. If the reviewer has additional opinions on what is unnecessary, we would be happy to consider them.

3. In methods, subsections could be added to conceptual framework since the description is lengthy. And difficult to follow the flow of the manuscript. Subsections could include: Policy interventions available, possible transitions, costs and benefits of intervention, cost-benefit analysis, process of actually performing the analysis, advantages and limitations.

Response: Thank you for these suggestions. We have included sub-sections in the ‘Methods’ section of the revised manuscript.

4. In discussion, comparing BAR-HAP tool with other similar energy policy-based tool would help. [e.g. (LEAP-IBC) tool to assess air quality and climate co-benefits, GridPIQ/Combined Heat and Power (CHP) Energy and Emissions Savings Calculator] that help to assess the effect of energy transition.

Response: Thank you for pointing us to other energy policy-based tools. In the revised manuscript, we have included a short section in the ‘Introduction’ section (#1) comparing the BAR-HAP Tool to these other tools. We feel this is valuable to include in the introduction because it helps to highlight and clarify the contribution of the work. See Pp. 4-5: “BAR-HAP is very different from other energy sector planning models, such as the Long-range Energy Alternatives Planning-Integrated Benefits Calculator (LEAP-IBC) and the Combined Heat and Power (CHP) Energy and Emissions Savings Calculator. LEAP-IBC, for instance, helps governments compute the greenhouse gases and short-lived climate pollutants and develop mitigation scenarios [21]. The CHP Energy and Emissions Savings Calculator meanwhile quantifies and “compares the estimated fuel consumption and air pollution emissions (CO_2_e, SO_2_ and NO_X_) of a CHP system and comparable separate heat and power system (e.g. grid power)” [22]. In contrast, the BAR-HAP Tool calculates the private net benefits (time savings, fuel savings, morbidity reductions and mortality reductions) and social net benefits (all private net benefits, along with community spillovers for health benefits, climate and environmental benefits) of potential cooking transitions within a given context, under real-world policy interventions. As became evident during piloting in Nepal, the tool facilitates interactions between relevant stakeholders – researchers, policy-makers outside of the health sector, and implementers of clean energy interventions – because it allows calculation of the government, household, and broader societal costs and benefits of improved cooking policies. Its aim is to support decision-making on ways to accelerate clean energy transitions at national or sub-national scales, by linking technology adoption and outcomes to the effectiveness of specific policy interventions.”

5. In figure 2 if available, to include what the predicted uptake for each intervention in year 1,2, 3…., or it could be included in a Table

Response: The requested information is provided on p.16 and would not easily fit in figure 2 because it can be modified by users: “In the default set-up, the initial five years are treated as a ramp up period, during which the intervention planning proceeds for two years without generating benefits, followed by three years over which a third of the target population is added in each year. Starting in year 6, then, the full target population is assumed to be affected. Alternatively, the policy interventions can be considered fully implemented starting in the first year, in which case benefits begin immediately.”

6. In page 14, the paragraph before Figure 3, ‘The cost effectiveness ratios (CER) range between about 674 and 869 US$/DALY avoided across subsidy levels’. Is there any data when the intervention would start becoming benefit (positive) independent of the DALY avoided? That is, when could we expect the net social benefit to equalize the cost invested, at 70% uptake.

Response: We have amended this section to specify the breakeven subsidy level, but note that this is not in reference to the public cost, but rather the overall social net benefits. Please see p. 21:

“At the same time, benefits also increase with adoption, and exceed costs in present value terms at all subsidy levels above 61%, which is the breakeven subsidy level from a social perspective.”

7. In Figure 4, the colors are very similar. If possible, to change to more distinct colors.

Response: Thank you for pointing this out. We have revised Figure 4 now to include more distinct colors.
